# The gastric mucosal-associated microbiome in patients with gastric polyposis

**DOI:** 10.1038/s41598-018-31738-2

**Published:** 2018-09-14

**Authors:** Rongrong Ren, Zikai Wang, Huaibo Sun, Xuefeng Gao, Gang Sun, Lihua Peng, Bin Yan, Yunsheng Yang

**Affiliations:** 1 Department of Gastroenterology and Hepatology, the Chinese PLA General Hospital, the Chinese PLA Medical College, Beijing, China; 2 Institute of Soil Science, Chinese Academy of Sciences, Nanjing, China; 3 Shenzhen University General Hospital, Shenzhen, China; 4 Shenzhen University Clinical Medical Academy, Shenzhen, China

## Abstract

The characteristics of the gastric microbiota in patients with gastric polyposis (GP) remain unclear. Given this we collected gastric antrum and gastric body biopsies from healthy controls (HC.A and HC.B group) and gastric antrum, gastric body and polyp biopsies from patients with multiple gastric polyps (GP.A, GP.B and GP.P group) for 16S rDNA sequencing. The results showed that the diversity of the gastric flora in the GP group was significantly lower than that of the HC group. The gastric flora composition of the GP group was significantly different from the HC group. However, flora diversity and compositions in different parts of the stomach (gastric antrum, gastric body or polyp tissue) were not significantly different. *H. pylori* abundance could influence the composition of gastric microbiota. Meanwhile, patients with fundic gland polyps (FGPs) and those with hyperplastic polyps (HPs) had considerably similar gastric bacterial compositions. We constructed a microbial dysbiosis index (MDI) based on the gastric microbiota at the genus level as a predictive model, and it was able to distinguish between individuals in the GP and HC groups. These findings showed that intragastric flora dysbiosis may be closely related to the occurrence and development of gastric polyps.

## Introduction

Gastric polyps are limited epithelial protrusions of the epithelial mucosa, and the most common pathological types are gastric fundic gland polyps (FGPs) and hyperplastic polyps (HPs)^1,2^. While their overall prevalence appears to have increased in the past thirty years^3^, the diagnosis and treatment of gastric polyps have attracted attention. However, their etiology, biological characteristics and long-term impact on the body remain unclear. There are many potential factors associated with the development of gastric polyps, including genes, bile reflux, Helicobacter pylori infection, and long-term use of proton pump inhibitors (PPIs)^4,5^. However, direct evidence for the involvement of these factors is lacking, and there is little new information regarding gastric polyps. In recent years, studies of microbial and human health and disease have begun to use metagenomic principles and high-throughput sequencing analysis, which have become popular research tools. However, most studies are based on gut microbe and focus on the relationship between gut microbes and intestinal and metabolic diseases. Few researchers have paid attention to the role of the microbiota in gastric diseases. The stomach is a special area with regards to the gastrointestinal microbiota, as it has its own unique ecological environment and characteristic microbial communities due to the secretion of gastric acid. Several existing studies on intragastric flora based on high-throughput sequencing have confirmed the existence of complex flora in the stomach^6,7^. According to these studies, there are large numbers of other microorganisms in addition to *H. pylori* in the stomach. In addition, gastric microbial dysbiosis plays an important role in gastric carcinogenesis^7^, but no studies have investigated the relationship between gastric polyps and the stomach flora. Some studies have confirmed that the intestinal flora and diversity in patients with colorectal cancer and colon polyps are altered, and the occurrence and development of colorectal cancer may be related to the intestinal flora^8,9^. More remarkable, one recent study showed an unexpected link between early colon neoplasia and tumorigenic bacteria based on patients with familial adenomatous polyposis^10^. However, we still do not know much about the characteristics of the gastric flora in patients with gastric polyps and their differences from those in healthy people.

In this study, we analyzed the gastric bacterial community composition and diversity characteristics of patients with gastric polyps. In addition, we constructed a microbial dysbiosis index (MDI) model which was able to predict gastric polyps based on the bacterial profiles of gastric.

## Results

### Patient samples

A total of 30 HCs and 30 GP patients were included in the study. The age and sex of the subjects were not significantly different between the two groups (P > 0.05) (Supplementary Table 1). In total, 148 eligible gastric mucosal biopsy samples were included in this study (Supplementary Table 2), including 30 gastric antrum samples (HC.A) and 29 gastric body samples (HC.B) from individuals in the HC group and 30 gastric antrum tissues (GP.A), 29 gastric body tissues (GP.B) and 30 gastric polyp tissues (GP.P) from patients in the GP group. The polyp tissues of the 30 patients with multiple gastric polyps were divided into two subgroups according to the pathological polyp type: 20 FGPs and 10 HPs. The infection rates of *H. pylori* as determined by a rapid urea test (RUT) in the different groups are shown in Supplementary Table 3. None of the healthy subjects were positive for *H. pylori* by RUT. In the GP group, the *H. pylori* infection rate was 15.4% (4/26). Moreover, *H. pylori* was detected by sequencing in all samples, and all subjects in both groups were positive for *H. pylori* based on this testing method. The mean value of *H. pylori* relative abundance was 0.139 ± 0.210 in the HC group and 0.0627 ± 0.208 in the GP group (P < 0.05).

### The general composition of the gastric mucosal-associated bacterial microbiota

**Figure 1:**
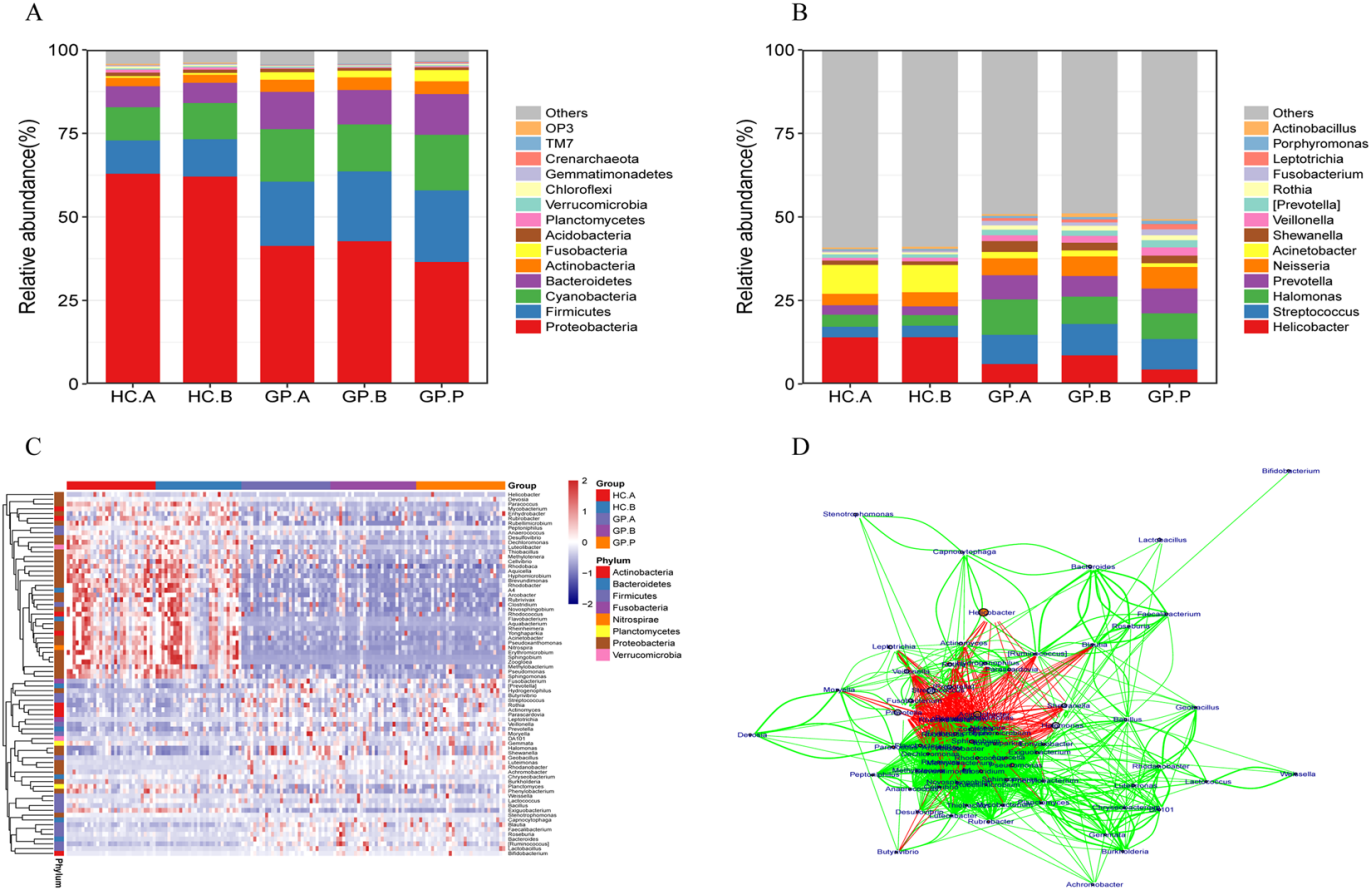
General characteristics of the gastric mucosal bacterial community composition. Relative abundances of the (**A**) most common phyla and (**B**) genera present in all samples among the study groups. The rare and unclassified taxa are summarized as “Others”. (**C**) Heatmap of the core genera in all samples of the HC and GP groups; the heatmap is color-coded based on row z scores and column clustered by an unweighted pair-group method with arithmetic means. (**D**) Network of core genera in all samples of the HC and GP groups. HC: healthy control; GP: gastric polyposis; (**A**) gastric antrum; (**B**) gastric body; P: gastric polyp.

Libraries were constructed for all samples by amplification of the V4 region of the bacterial 16S rDNA gene. A total of 4,558,009 raw reads were obtained after quality filtering, with an average of 30,797 for each sample. A total of 68 bacterial phyla were identified in all gastric biopsy samples. To analyze the difference in the gastric mucosal flora at different sites, we divided the samples into the HC.A, HC.B, GP.A, GP.B and GP.P groups. The dominant bacteria at the phyla level of these five groups were similar. The most common phyla in the stomach are shown in Fig. 1A, and they accounted for 98% of the sequencing data. The top ten phyla included *Proteobacteria*, *Firmicutes*, *Cyanobacteria*, *Bacteroidetes*, *Actinobacteria*, *Fusobacteria*, *Acidobacteria*, *Planctomycetes*, *Verrucomicrobia* and *Chloroflexi*. The HC tissue groups had a much higher abundance of *Proteobacteria* but a lower abundance of *Firmicutes*, *Bacteroidetes, Actinobacteria*, *Fusobacteria* and *Acidobacteria* (Supplementary Table 4).

**Figure 2:**
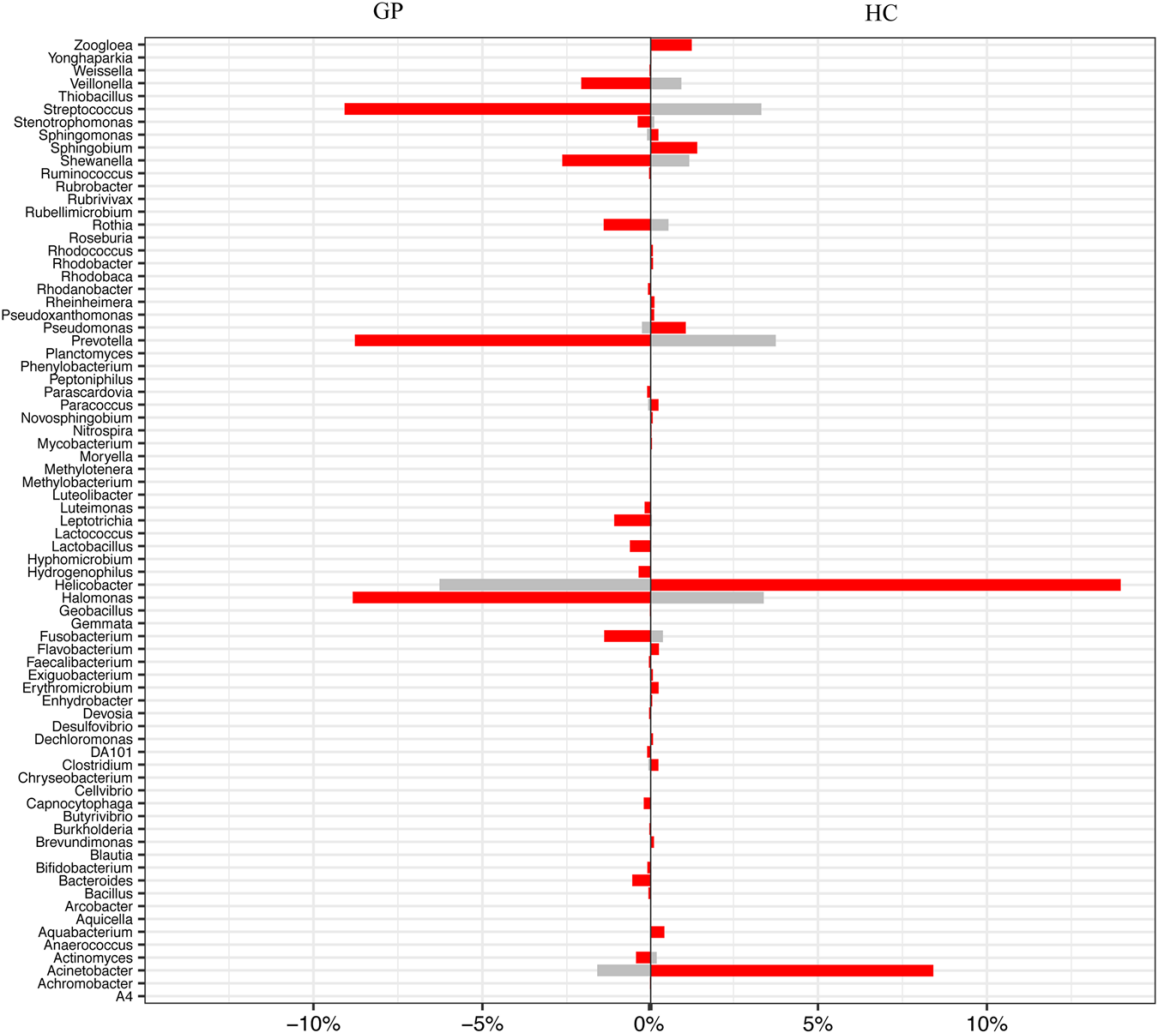
The significantly predominant bacterial taxa in the GP and HC groups. Wilcoxon rank-sum tests of the relative abundances, with P < 0.05 and detected in at least 70% of the samples were shown. HC: healthy control; GP: gastric polyposis.

Furthermore, 598 genera were identified in the stomach, and the top ten were *Helicobacter*, *Streptococcus*, *Halomonas*, *Prevotella*, *Neisseria*, *Acinetobacter*, *Shewanella*, *Veillonella*, *Rothia*, and *Fusobacerium* (Fig. 1B). In addition, we detected significantly different core genera in at least 70% of the samples, and the *p* values were less than 0.05 by variance analysis among these five groups, as shown in the core genus heatmap (Fig. 1C), and their relationships are shown in the network figure (Fig. 1D) conducted with spearman rank correlation coefficient. The network figure exhibited an antagonistic relationship between HC-enriched genera and GP-enriched genera, and a synergistic relationship among HC-enriched genera or among GP-enriched genera. Moreover, GP-enriched and HC-depleted genera included *Veillonella*, *Prevotella*, *Lactobacillus*, *Streptococcus*, *Fusobacterium*, *Rothia*, *Bacteroides*, *Actinomyces*, *Halomonas*, and *Shewanella*, while GP-depleted and HC-enriched genera included *Helicobacter*, *Acinetobacter*, *Sphingobium*, *Pseudomonas*, *Nitrospira* and *Rhodococcus* (Fig. 2).

### Alpha diversity of the gastric bacterial microbiota in different groups

**Figure 3:**
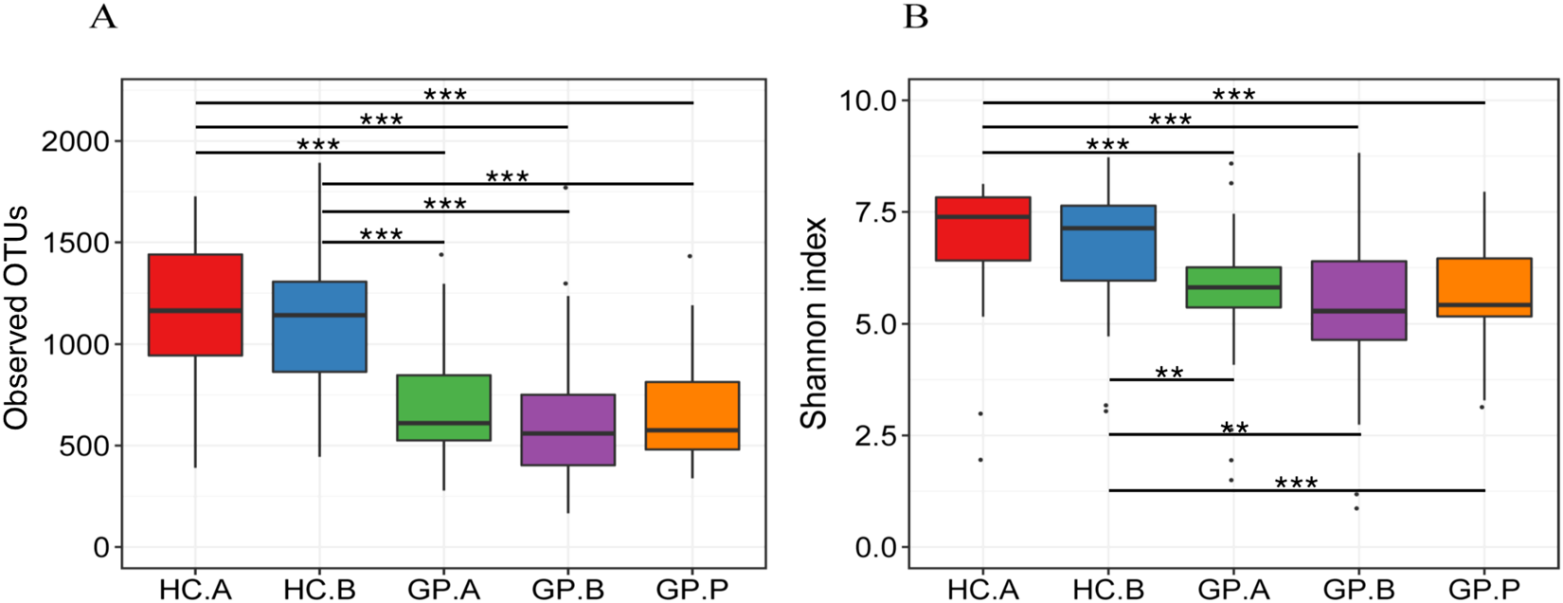
Alpha diversity of gastric bacterial communities is analyzed and compared among the HC and GP groups. (**A**) The observed OTUs and (**B**) the Shannon diversity index measured in the study groups. HC: healthy control; GP: gastric polyposis; (**A**) gastric antrum; (**B**) gastric body; P: gastric polyp. **P < 0.01; ***P < 0.001.

The average OTU numbers were 1,151 ± 338, 1,108 ± 336, 688 ± 277, 653 ± 360, and 674 ± 283 in the HC.A, HC.B, GP.A, GP.B and GP.P groups, respectively. The diversity of the gastric bacterial microbiota in the HC.A and HC.B groups was similar, as was that in the GP.A, GP.B and GP.P groups. However, the gastric bacterial diversity in the GP tissue groups was significantly lower than that of the HC tissue groups as measured by observed OTUs and the Shannon diversity index (Fig. 3A,B).

### Beta diversity of the gastric bacterial microbiota in different groups

**Figure 4:**
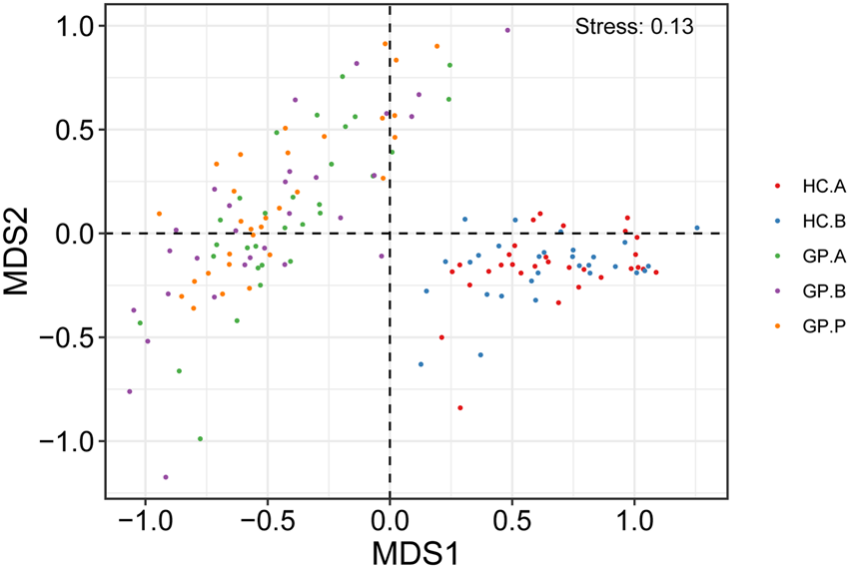
Beta diversity of gastric bacterial communities of the HC and GP groups. NMDS ordination plot of gastric bacterial communities based on the Bray-Curtis distance metric. HC: healthy control; GP: gastric polyposis; (**A**) gastric antrum; (**B**) gastric body; P: gastric polyp.

**Figure 5:**
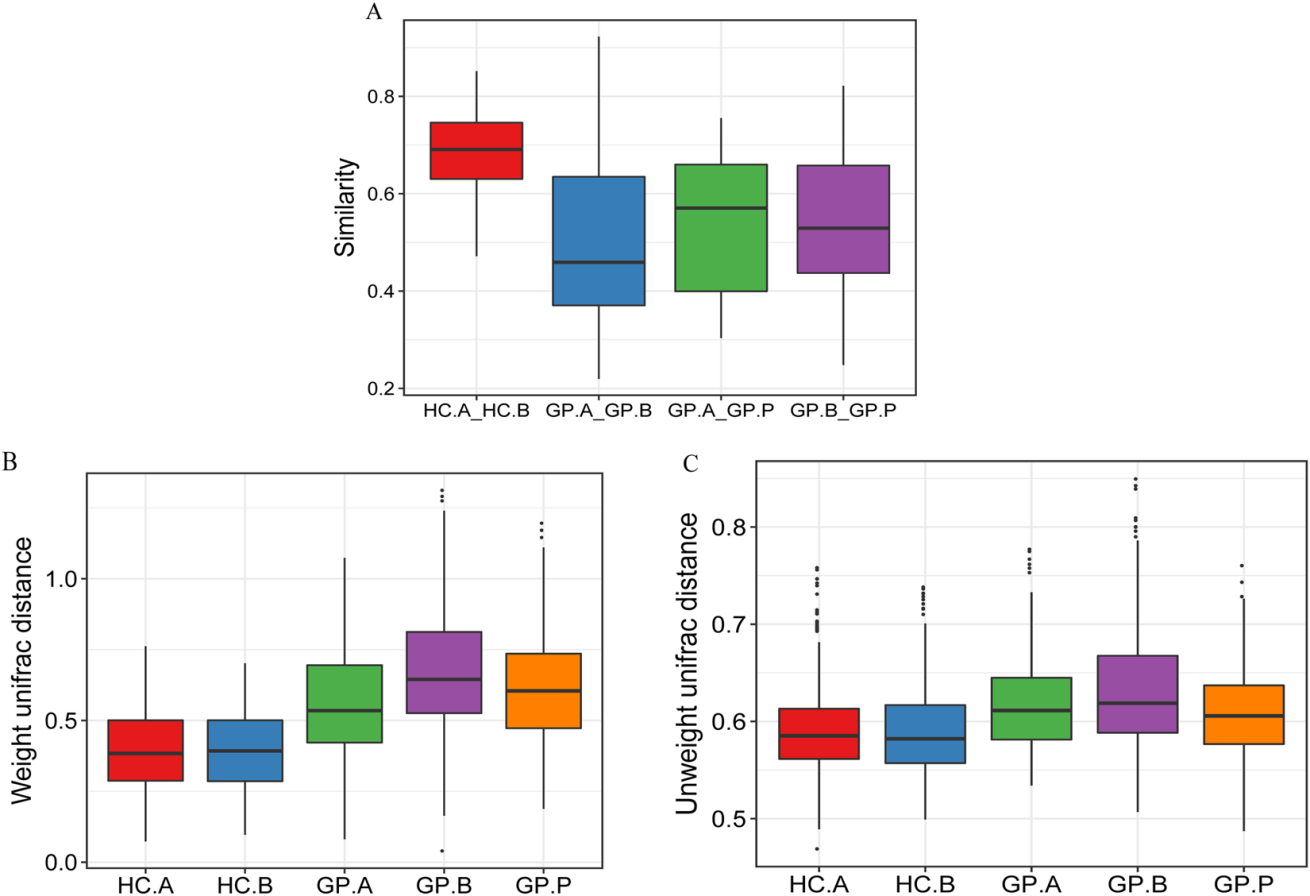
Community similarity analysis. (**A**) Similarity index of gastric bacterial communities in the biopsy samples between HC groups and GP groups respectively; (**B**) weighted and (**C**) unweighted unifrac distance of gastric bacterial communities in the samples among HC.A, HC.B, GP.A, GP.B and GP.P. HC: healthy controls; GP: gastric polyposis.

**Figure 6:**
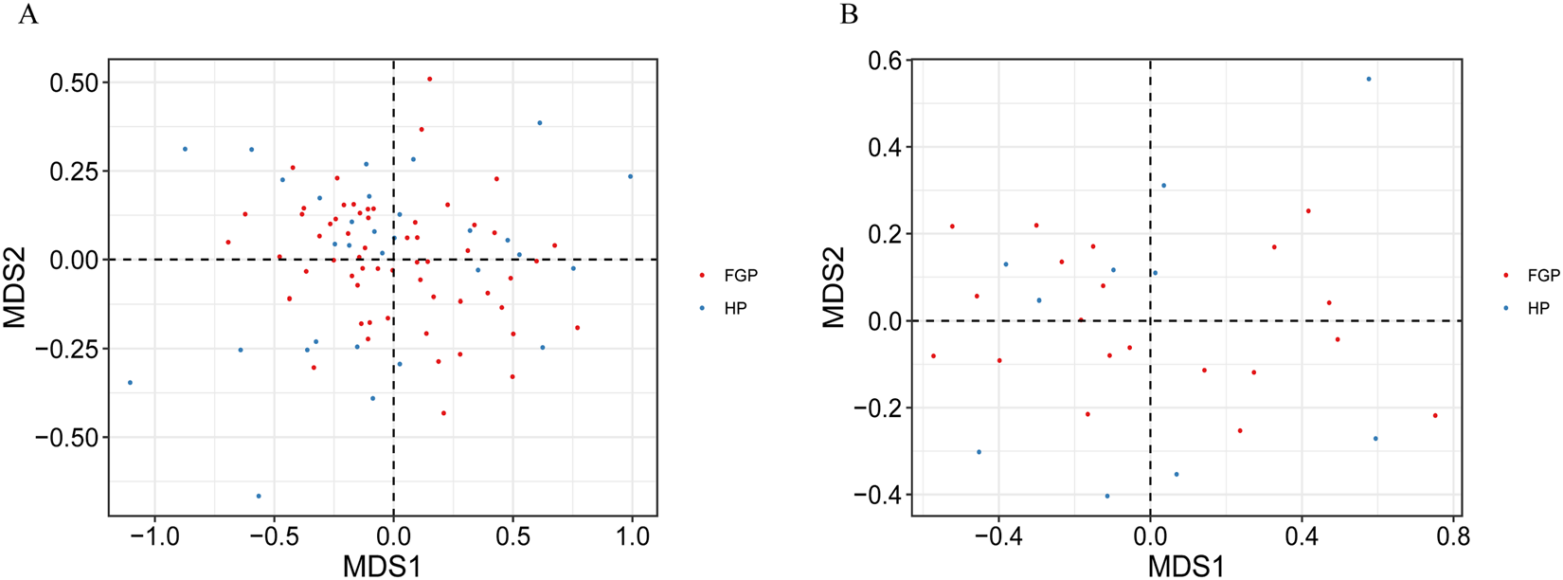
Beta diversity of gastric bacterial communities of the FGP and HP groups. NMDS analysis of gastric bacterial communities from the 30 patients with gastric polyposis (**A**) from all biopsy samples among the FGP and HP groups, and (**B**) from only the polyp samples. FGP: Fundic gland polyp; HP: hyperplastic polyp.

Non-metric multidimensional scaling (NMDS) analysis and weighted and unweighted Principal Coordinates Analysis (PCoA) indicated that the HC and GP groups clustered separately in terms of gastric microbiota composition. The HC.A and HC.B groups showed similar gastric microbiota compositions, as did the GP.A, GP.B and GP.P groups (Fig. 4 and Supplementary Fig. S1). However, the similarity between the HC.A and HC.B groups was much higher than the similarity among the GP.A, GP.B and GP.P groups (Fig. 5A); and weighted and unweighted unifrac distance showed that there was a larger degree of intra-subject variability in gastric bacterial diversity (which means the differences of gastric bacterial diversity among the subjects who are in the same group) among GP patients than in healthy subjects (Fig. 5B,C). In addition, we analyzed the gastric bacterial composition of patients with FGPs and those with HPs. NMDS analysis and unweighted PCoA could not distinguish the two groups significantly, while weighted PCoA showed a considerable difference between the two groups (Fig. 6A,B and Supplementary Figs S2 and S3).

### The influence of *H. pylori* on the diversity and composition of the bacterial microbiota in the stomach

**Figure 7:**
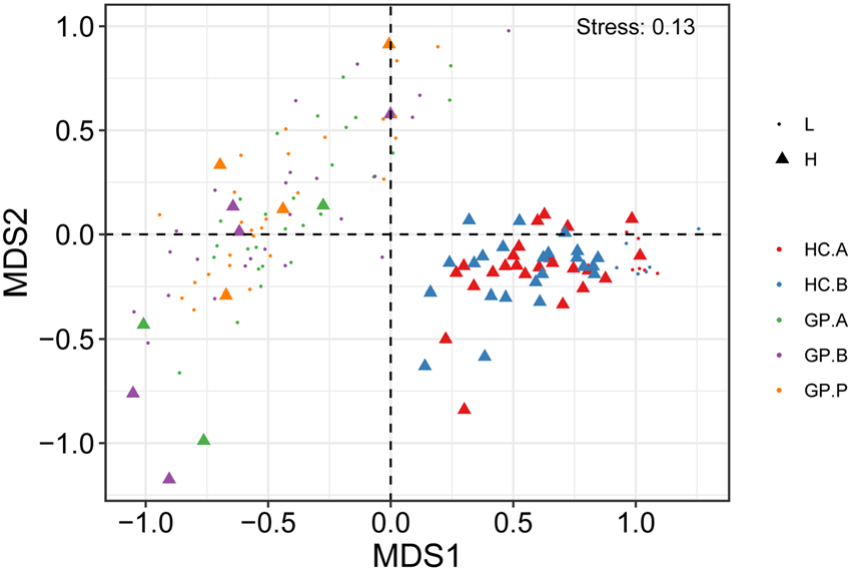
Influence of *H. pylori* on the diversity and composition of the bacterial microbiota in the stomach. NMDS analysis of gastric bacterial communities in the samples among the HC.A, HC.B, GP.A, GP.B and GP.P groups based on the abundance of *H. pylori*. L: low *H. pylori* abundance <1%; H: high *H. pylori* abundance ≥1%. HC: healthy control; GP: gastric polyposis; (**A**) gastric antrum; (**B**) gastric body; P: gastric polyp.

In a previous study, gastric mucosal biopsies with <1% *H. pylori* abundance were classified as *H. pylori*-negative, while >1% were classified as *H. pylori*-positive^7^. However, this cutoff value of *H. pylori* sequence percentage was based on clinically positive *H. pylori* status^11^. And there were still *H. pylori* sequence in those *H. pylori*-negative samples. As we will explore the influence of *H. pylori* according to its sequences, we classified the samples into two groups: *H. pylori*-low group (*H. pylori* abundance <1%), and *H. pylori*-high group (*H. pylori* abundance ≥1%). NMDS analysis and weighted and unweighted PCoA showed that the gastric microbiota composition was significantly different between subjects with high *H. pylori* abundance and subjects with low *H. pylori* abundance (Fig. 7 and Supplementary Fig. S4). The *H. pylori*-high group had a much higher abundance of *Proteobacteria* and *Nitrospirae*, but a lower abundance of *Firmicutes* and *Fusobacteria* than *H. pylori*-low group in phylum level (Supplementary Fig. S5). And in genera level, the abundances of *Helicobacter*, *Acinetobacter*, *Zoogloea* and *Sphingobium* were much higher in *H. pylori*-high group, while the abundances of *Shewanella*, *Bacteroides*, *Hydrogenophilus* and *Lactobacillus* were much higher in *H. pylori*-low group (Supplementary Fig. S6). In addition, we investigated the effect of *H. pylori* on the distinction of FGP and HP, and the results showed that these two groups could not be distinguished based on the *H. pylori* abundance (Supplementary Figs S7 and S8).

### The microbial dysbiosis index as a predictive model

**Figure 8:**
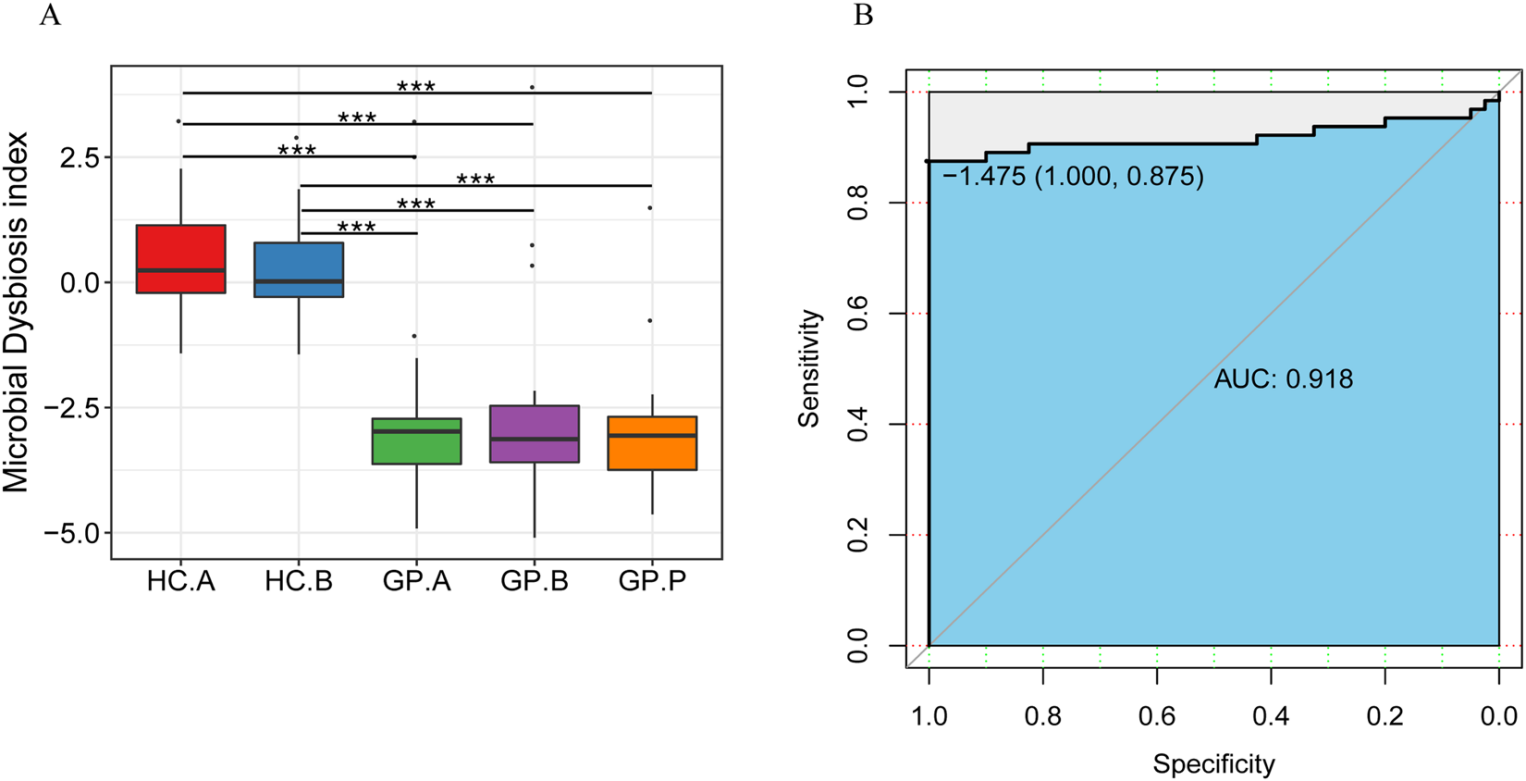
(**A**) Predicting gastric polyposis with MDI. (**A**) MDI comparison among the study groups. (**B**) ROC curve analysis to evaluate the discriminatory potential of the MDI in the discovery cohort of the HC group and GP group. HC: healthy control; GP: gastric polyposis; (**A**) gastric antrum; (**B**) gastric body; P: gastric polyp. ***P < 0.001.

To illustrate the diagnostic value of the gastric mucosal-associated bacterial microbiota in GP, we constructed a microbial dysbiosis index (MDI) model based on genera appeared in 70% (104/148) of all the samples. The performance of the predictive model was defined as the log of [total abundance of the core genera increased in the HC group relative to the GP group] over [total abundance of the core genera decreased in the HC group relative to the GP group]^12^, using the core genera increased in the HC group, including *Helicobacter* and *Acinetobacter*, and the core genera decreased in the HC group, including *Prevotella*, *Streptococcus*, *Veillonella*, *Neisseria*, *Halomonas*, and *Shewanella* (Fig. 8A). The core genera that were significantly different between the HC and GP groups were chosen from the top ten genera in terms of abundance. This model could distinguish between the HC and GP groups. However, it could not distinguish between the HC.A and HC.B tissue groups or between the GP.A, GP.B and GP.P tissue groups (Fig. 8A). In general, the predictive model constructed by the MDI exhibited a high discriminatory potential in the discovery analysis of the HC and GP groups, with an AUC of 91.8% (Fig. 8B). We then verified this index using the rest 30% samples (44/148) and the accuracy is 93.2%, the precision is 100% and recall is 88%.

## Discussion

In recent years, high-throughput sequencing and bioinformatics analysis technology have been used to study various parts of the human body, especially the structure and function of microbial communities in the digestive tract, while only a few studies have evaluated the microbial communities in the stomach. It was traditionally thought that the human stomach was not suitable for the growth and reproduction of microorganisms due to the presence of gastric acid and other antimicrobial agents. With the discovery of *H. pylori* and other *Helicobacter* species in the stomach, and the application of high-throughput sequencing in recent years, the study of gastric flora has confirmed that the stomach is a special microbial environment, similar to the gut^13^. However, in recent years, research on intragastric flora has mainly focused on the changes in the intragastric flora in gastric cancer, atypical hyperplasia, intraepithelial neoplasia and other precancerous lesions. Explorations of the composition of the gastric flora and the pathogenesis of gastric polyps from the perspective of gastrointestinal microecology have not been reported. The present study attempted to characterize the gastric mucosal bacterial microbiota in GP, to identify the significant related phylotypes and to construct a predictive model for the diagnosis of GP based on bacterial markers.

Our study found that patients with gastric polyps and healthy people had similar dominant bacteria at the phylum level, with the top phyla being *Proteobacteria*, *Firmicutes*, *Cyanobacteria*, *Bacteroidetes* and *Actinobacteria*; this result was partly consistent with one previous study showing that the majority of sequences in the gut microbiome were assigned to the *Proteobacteria*, *Firmicutes*, *Actinobacteria*, *Bacteroidetes* and *Fusobacteria* phyla^14^. At the genus level, *Helicobacter*, *Streptococcus*, *Prevotella*, *Neisseria*, *Rothia*, and *Fusobacterium* were highly abundant, which is similar to previous studies^14–17^. Our study also showed that, the intragastric flora diversity was significantly lower in the GP group than that in the HC group. Moreover, the composition of the intragastric flora in the two groups had significant differences. These findings showed that patients with gastric polyps have significantly different stomach flora than healthy people, and intragastric flora dysbiosis may be closely related to the occurrence and development of gastric polyps.

A recent study showed that gastric cancer patients had a higher abundance of *Streptococcus* and *Fusobacterium* species and a lower abundance of *Acinetobacter* species^7^. We found that gastric bacteria that were enriched in gastric cancer patients were also highly abundant in patients with gastric polyps, while gastric bacteria with low abundance in gastric cancer patients also had low abundance in patients with gastric polyps, suggesting that these bacteria may be associated with the occurrence of gastric polyps and the progression of gastric polyps to gastric cancer. However, there was also one study that was inconsistent with our conclusion. This study showed that in patients with gastric cancer, *Rhodococcus, Clostridium, Lactobacillus, Citrobacter* and *Achromobacter* were highly abundant, and *Helicobacter, Streptococcus, Neisseria* and *Prevotella* had low abundances^6^.

In addition, we found that both the HC and GP groups showed a high abundance of halophilic bacteria, such as Halomonas and Shewanella, which was not consistent with previous studies^6,14–17^. This may be related to the consumption of salty or pickled foods by Chinese individuals, as previous studies mainly focused on western countries. However, the abundance of halophilic bacteria in the GP group was significantly higher than that in the HC group, suggesting that eating preserved food or high-salt food may be a risk factor for gastric polyps.

Previous studies have suggested that the stomach flora compositions of the gastric body, gastric antrum, lesions and non-mucosal lesions are similar^7,14,18^, but this result is controversial^19,20^. Our study found that in both the HC group and the GP group, the flora compositions in different sites (gastric antrum, gastric body and gastric polyps) were similar, and the flora diversity between different tissue subgroups of the same group were also similar, indicating that the composition of stomach bacteria may not be related to the location or even the lesion. This is consistent with previous studies. Our study also found that although there was no significant difference between the gastric antrum and the gastric body or between the normal and diseased mucosa, the flora composition difference in different sites of GP group was larger than the difference observed in HCs. In addition, the individual differences in the GP group were also larger than the individual differences in the HC group. This indicates that the intragastric flora in patients with gastric polyps was more variable than that in HCs, which further confirmed that gastric polyps in patients are associated with gastric flora dysbiosis.

In our study, all subjects in the HC group were *H. pylori*-negative in the stomach as determined by a routine clinical test. However, after high-throughput sequencing, we found that *H. pylori* DNA was detected in all subjects in both groups. This is primarily because the high-throughput sequencing method based on bacterial 16S rDNA is more sensitive than the traditional clinical *H. pylori* detection methods, and it can detect *H. pylori* sequences with extremely low abundance in the stomach^14^. Our study showed that the gastric microbiota compositions were different according to *H. pylori* abundance, especially that *H. pylori*-high subjects had a much higher relative abundance of *Helicobacter* than *H. pylori*-low subjects. Notably, the *H. pylori* abundance in the stomach could affect the composition of the intragastric flora. This conclusion was similar to previous studies^21,22^. In addition, our study showed that the *H. pylori* abundance in the HC group was significantly higher than that in the GP group, however, the *H. pylori* abundance could not distinct FGP and HP. These results suggested that the presence of *H. pylori* may be inversely associated with the occurrence of FGP and HP. In other words, the presence of *H. pylori* may be a protective factor for these benign gastric polyps. This result was consistent with previously published data which reported that the presence of fundic gland polyps was inversely correlated with *H. pylori* infection^3,23^.

In this study, MDI was calculated based on the significantly different genera in the HC and GP groups, and the HC and GP groups could be distinguished clearly with the AUC reaching 0.918, and the accuracy and precision of verification reached 93.2% and 100%, respectively. The MDI index could be used as a biomarker of microbial indicators of GP in future clinical applications and it may predict people who have the tendency of having GP. However, there was only a small validation set of data used to assess the performance of this index in the present study, and a larger number of samples are needed to validate this predictive index.

There were several limitations in this study. The population size was not large, and the number of samples assigned to the subgroups was relatively small; thus, a larger number of samples are required to validate the results of this study. Analysis of 16S rDNA can only discriminate to the genus level with limited accuracy, and more specific sequencing and analysis techniques are required to determine which bacterial species or strains are involved in the pathogenesis of GP. In addition, this study only analyzed the stomach bacteria, and whether the stomach contains fungi or viruses and the effects of their changes require further study.

## Conclusions

The diversity of the gastric flora in patients with gastric polyps was significantly lower than that of healthy people, and the intragastric bacterial composition between the two groups was significantly different. However, flora compositions in different parts of the stomach (gastric antrum, gastric body or polyp tissue) were similar. *H. pylori* could alter the structure of the gastric microbiota. The MDI we designed was able to differentiate between the healthy and polyp groups, but further validation with a larger population is needed.

## Material and Methods

### Participants

This study was conducted at the Chinese PLA General Hospital, Beijing, China. From May 2012 to December 2013, consecutive patients with gastric polyposis (GP) were recruited. All patients were diagnosed by gastroscopy and pathology at the first visit and did not receive any treatment. As healthy controls (HCs), we selected subjects that had no gastrointestinal symptoms, tested negative for *H. pylori* with the rapid urease test (RUT), and had normal gastric mucosal morphology based on a physical check-up. Demographics, *H. pylori* infection status and medications were assessed. This study was carried out according to the Helsinki Declaration and written informed consent was obtained from all subjects. This study was approved by the ethics committee of the Chinese PLA General Hospital and was registered with the WHO ICTRP (ID: ChiCTR-OCC-12002573).

To be eligible for participation in this study, all subjects were required to meet the following inclusion criteria: (1) adult male or female; (2) of Han nationality from the northern areas of China; (3) able and willing to provide gastric mucosal biopsy samples; and (4) able and willing to provide signed informed consent. GP patients were confirmed with histological analysis of gastric mucosal biopsy samples. Subjects who met any of the following criteria were excluded from this study: (1) having taken antibiotics, proton pump inhibitors (PPIs), non-steroidal anti-inflammatory drugs (NSAIDs), probiotics, prebiotics, corticosteroids, chemotherapy drugs or any other drugs that affect the gastrointestinal microbiota within the last month; (2) diagnosed with acute or chronic pulmonary, cardiovascular, hepatic or renal disorders; (3) positive for human immunodeficiency virus (HIV), hepatitis B virus (HBV), hepatitis C virus (HCV) or hepatitis V virus; (4) a history of major surgery; and (5) pregnant or lactating.

### Collection of gastric mucosal biopsy samples

All subjects were fasting and were not taking drugs that affect the gastrointestinal flora when they underwent gastroscopy. The gastric mucosal biopsy samples were obtained with standard and sterilized endoscopy forceps. To avoid contamination, new sterilized endoscopy forceps were used when taking a second biopsy from the same subject. For individuals in the HC group, two normal gastric mucosal biopsy samples from the gastric antrum and body were collected and labeled HC.A and HC.B, respectively. For patients in the GP group, two gastric mucosal biopsy samples from the normal gastric antrum and body were collected and labeled GP.A and GP.B, respectively, one polyposis tissue sample was collected and labeled GP.P, and polyposis was confirmed by histology. The biopsy samples were frozen in liquid nitrogen immediately, transferred to laboratory and stored at −80 °C.

### DNA extraction and PCR amplification

Total genome DNA from samples was extracted using the QIAamp DNA Mini Kit (QIAGEN, Valencia, CA, USA) combined with the bead-beating method^14^. The DNA concentrations of each sample were adjusted to 50 ng/μl for subsequent 16S rDNA genes analysis. The bacterial DNA was stored at –80 °C for sequencing.

16S rDNA genes of V4 region were amplified used universal primers (F: 5′-GTGCCAGCMGCCGCGGTAA-3′, R: 5′-GGACTACHVGGGTWTCTAAT-3′) with a 6-bp barcode. All PCR reactions (including denaturation, annealing and elongation) were carried out with Phusion® High-Fidelity PCR Master Mix (New England Biolabs). After electrophoresis of PCR products, samples with bright main strip between 400–450 bp were chosen for next mixing and purification with Qiagen Gel Extraction Kit (Qiagen, Germany). At last, sequencing libraries were generated and sequenced on an Illumina MiSeq PE-300 platform (Illumina, San Diego, USA). Barcodes and sequencing primers were trimmed before assembly.

### 16S rDNA gene sequencing data analysis

Paired-end reads were merged using FLASH (V1.2.7, http://ccb.jhu.edu/software/FLASH/)^24^ and raw tags were obtained. Then quality filtering on the raw tags was done with QIIME (V1.7.0, http://qiime.org/index.html)^25^ to obtain the high-quality clean tags^26^. These trimmed sequences were then chimera filtered, and singletons were discarded, and the sequences were assigned to operational taxonomic units (OTUs) (with a cutoff of 97% sequence identity for 16S rDNA) using the Uparse pipeline (V7.0.1001, http://drive5.com/uparse/)^27–29^. For each representative sequence of OTU was screened and aligned against the non-redundant SILVA database (version 123) using the RDP algorithm (V 2.2, http://sourceforge.net/projects/rdp-classifier)^30^. Alpha- and beta- analyses were performed using observed OTUs and Unifrac distances, respectively, as implemented in QIIME (V1.7.0).

### Statistical analysis

All statistical analysis was conducted with the program R software (version 3.4.1), using the packages vegan, stat, pROC. Spearman rank correlation coefficient was used to assess the correlation between taxonomic relative abundance. Significance differences in alpha diversity, beta diversity and taxonomy between groups were tested using Wilcoxon rank-sum test. Continuous data were presented as the mean ± SD and analyzed by the Student’s *t* test (*P* < 0.05). And categorical data were analyzed by the Chi-square test (P < 0.05). Similarity index were statistic between two different samples, based on counts at each sample. The formula is as follows:

$$similarity=\frac{2{C}_{ij}}{{S}_{i}+{S}_{j}}$$

C_ij_ is the sum of the lesser values for only those species in common between both sites. S_i_ and S_j_ are the total number of specimens counted at both sites.

PCoA was employed to determine differences between microbial communities using weighted and unweighted unifrac dissimilarity distance metric. NMDS analysis was performed with the Vegan package (version 2.4-4) in R software.

### Ethics approval

This study was approved by the Regional Ethical Review Board of the Chinese PLA General Hospital.

## Electronic supplementary material

Supplementary information
