# Supplementary material for: The gastric mucosal-associated microbiome in patients with gastric polyposis
Source: Sci Rep. 2018 Sep 14;8:13817. doi: 10.1038/s41598-018-31738-2 (PMC6138709; doi:10.1038/s41598-018-31738-2)
Supplement: Supplementary file 1 — Supplementary information [file 41598_2018_31738_MOESM1_ESM.pdf]

The gastric mucosal-associated microbiome in patients with gastric polyposis

Rongrong Ren,[1#] Zikai Wang,[1#] Huaibo Sun,[2#] Xuefeng Gao,[3,4] Gang Sun,[1] Lihua Peng,[1] Bin Yan,[1] Yunsheng Yang[1*]

[1] Department of Gastroenterology and Hepatology, the Chinese PLA General Hospital, the Chinese PLA Medical College, Beijing, China.

[2] Institute of Soil Science, Chinese Academy of Sciences, Nanjing, China.

[3] Shenzhen University General Hospital, Shenzhen, China.

[4] Shenzhen University Clinical Medical Academy, Shenzhen, China.

[#] These authors contributed equally to this work.

[*] **Correspondence**: Prof. Yunsheng Yang, Department of Gastroenterology and Hepatology, the Chinese PLA General Hospital, the Chinese PLA Medical College, Fuxing Road 28, 100853, Beijing, China. Email: sunny301ddc@126.com.

Supplementary table 1. Population characteristics

Characteristics	HC (n=30)	GP (n=30)	*P* value
**Age (yrs)**	45.6 ± 14.8	56.7± 14.2	0.824
**Gender (male/female)**	15/15	13/17	0.850

HC: healthy controls; GP: gastric polyposis; Comparison of the age between HC and GP groups was analyzed with Student's *t* test and comparison of the gender was by the *Chi-square* test.

Supplementary table 2. The eligible samples for sequencing among these groups

HC (n = 30)		GP (n = 30)	
HC.A	30	GP.A	30
HC.B	29	GP.B	29
		GP.P	30

HC: healthy controls; GP: gastric polyposis; A: gastric antrum; B: gastric body; P: gastric polyposis

Supplementary table 3. *H. pylori* characteristics in stomach among the HC and GP groups

Characteristics	HC (n = 30)	GP (n = 30)	*P* value
***H. pylori* clinical test (+/-)**	0/30	4/26	$P > 0.05$
***H. pylori* sequencing (+/-)**	30/0	30/0	$P > 0.05$
**Mean value of *H. pylori* relative abundance**	0.139±0.210	0.0627±0.208	$P < 0.05$

HC: healthy controls; GP: gastric polyposis; Statistical significance was determined by Wilcoxon-rank sum test.

Supplementary table 4. *P* values of Wilcoxon-rank sum test that used to compare the phyla among the HC and GP groups.

group1	group2	Proteobacteria	Firmicutes	Cyanobacteria	Bacteroidetes	Actinobacteria	Fusobacteria	Acidobacteria	Planctomycetes	Verrucomicrobia	Chloroflexi	Gemmatimonadetes	Crenarchaeota	TM7	OP3	Others
GP.A	GP.B	0.74635	0.8156	0.1529	0.5221	0.6570	0.1912	0.4743	0.9341	0.4467	0.5520	0.5930	0.8496	0.3460	0.9110	0.7123
GP.A	GP.P	0.2301	0.6865	0.3980	0.6022	0.9124	0.8430	0.9474	0.7082	0.8430	0.7412	0.6022	0.5944	0.0274	0.3523	0.3280
GP.A	HC.A	0.0000	0.0000	0.1381	0.0216	0.0096	0.0000	0.0019	0.0000	0.0115	0.0000	0.0002	0.2579	0.4761	0.0000	0.5039
GP.A	HC.B	0.0000	0.0015	0.6354	0.0113	0.0048	0.0000	0.0051	0.0000	0.0324	0.0000	0.0008	0.8319	0.4558	0.0000	0.8039
GP.B	GP.P	0.5419	0.5826	0.6035	0.3232	0.7236	0.2673	0.5027	0.9102	0.4289	0.4029	0.3778	0.6008	0.2299	0.4185	0.5826
GP.B	HC.A	0.0000	0.0200	0.6570	0.1002	0.2874	0.0003	0.0005	0.0000	0.0008	0.0000	0.0004	0.2165	0.0853	0.0000	0.2806
GP.B	HC.B	0.0000	0.0426	0.2519	0.0932	0.1536	0.0030	0.0016	0.0000	0.0053	0.0000	0.0006	0.9504	0.0815	0.0000	0.3952
GP.P	HC.A	0.0000	0.0001	0.8776	0.0061	0.0632	0.0000	0.0025	0.0000	0.0084	0.0000	0.0023	0.1579	0.0003	0.0000	0.0142
GP.P	HC.B	0.0000	0.0006	0.5320	0.0053	0.0364	0.0000	0.0108	0.0000	0.0337	0.0006	0.0046	0.5046	0.0005	0.0000	0.0491
HC.A	HC.B	0.8508	0.5826	0.2420	0.9460	0.4201	0.6900	0.5027	0.3944	0.3944	0.3944	0.4650	0.1476	0.9700	0.1244	0.6035

HC: healthy controls; GP: gastric polyposis; A: gastric antrum; B: gastric body; P: gastric polyp.

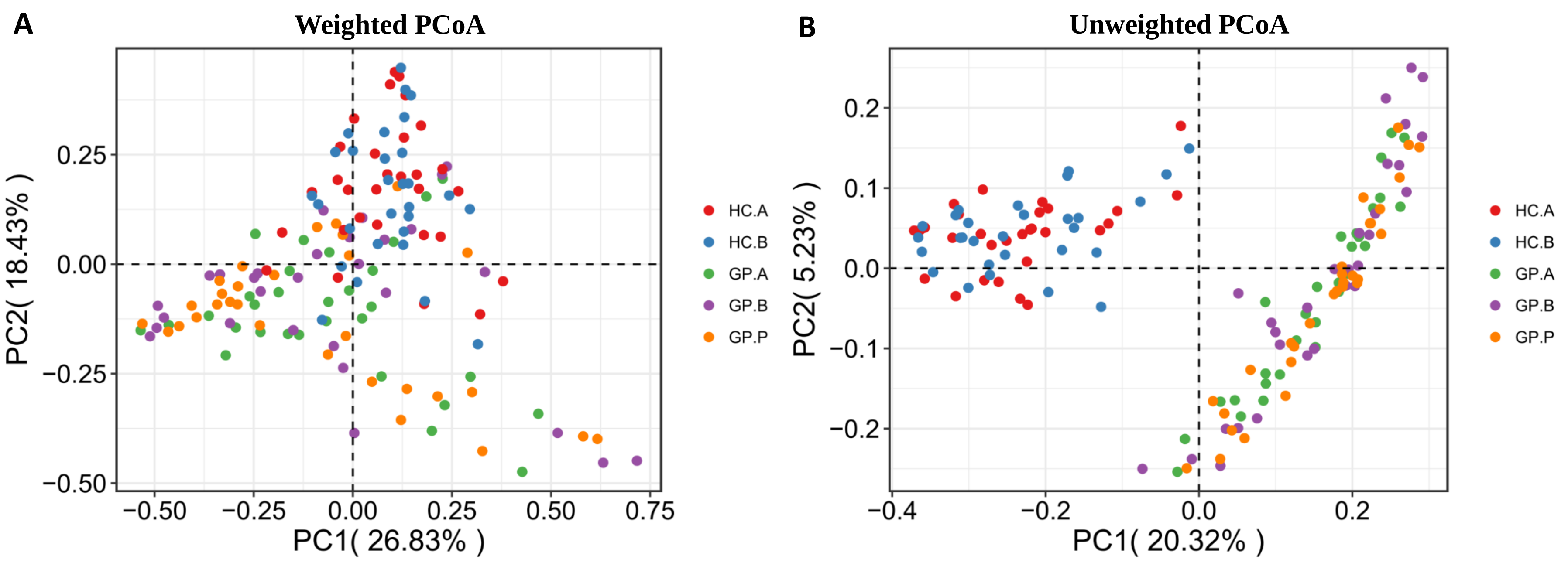

Supplementary figure S1. Beta diversity analysis of gastric bacterial communities in the samples among the HC and GP groups. (A) Weighted and (B) unweighted PCoA. HC: healthy control; GP: gastric polyposis; A: gastric antrum; B: gastric body; P: gastric polyp.

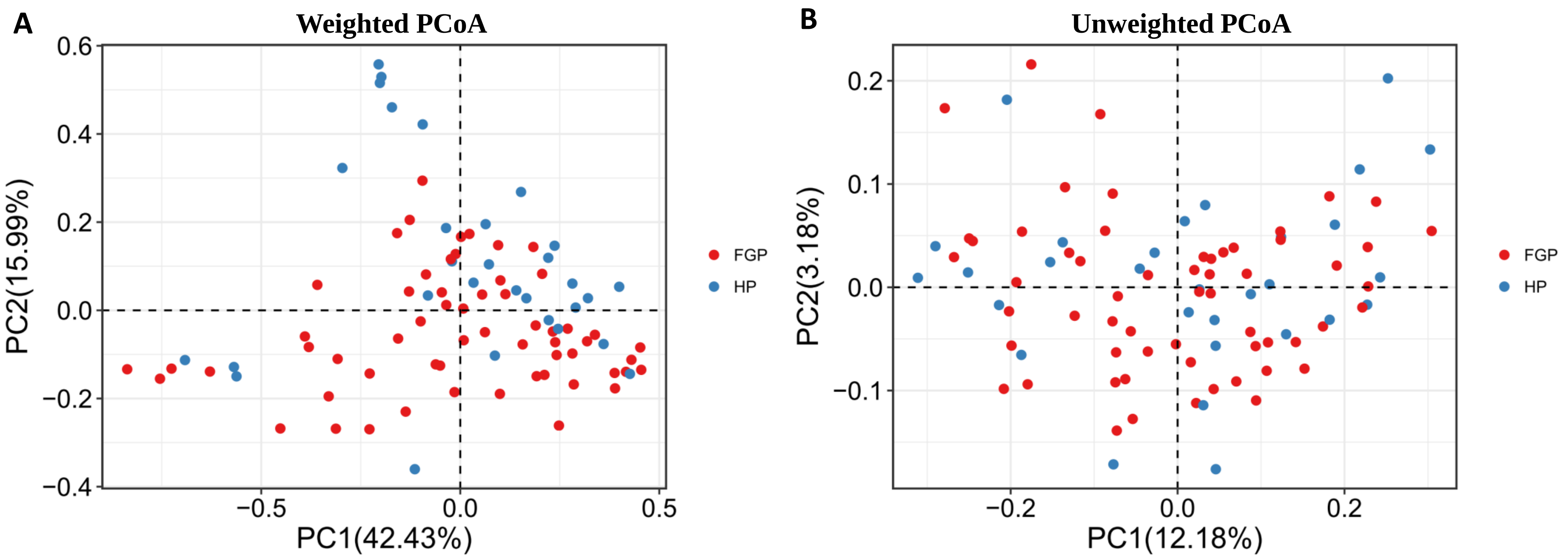

Supplementary figure S2. Beta diversity analysis of gastric bacterial communities in the samples between the FGP and HP groups of all the samples from the 30 patients with gastric polyposis. (A) Weighted and (B) unweighted PCoA. FGP: Fundic gland polyp; HP: hyperplastic polyp.

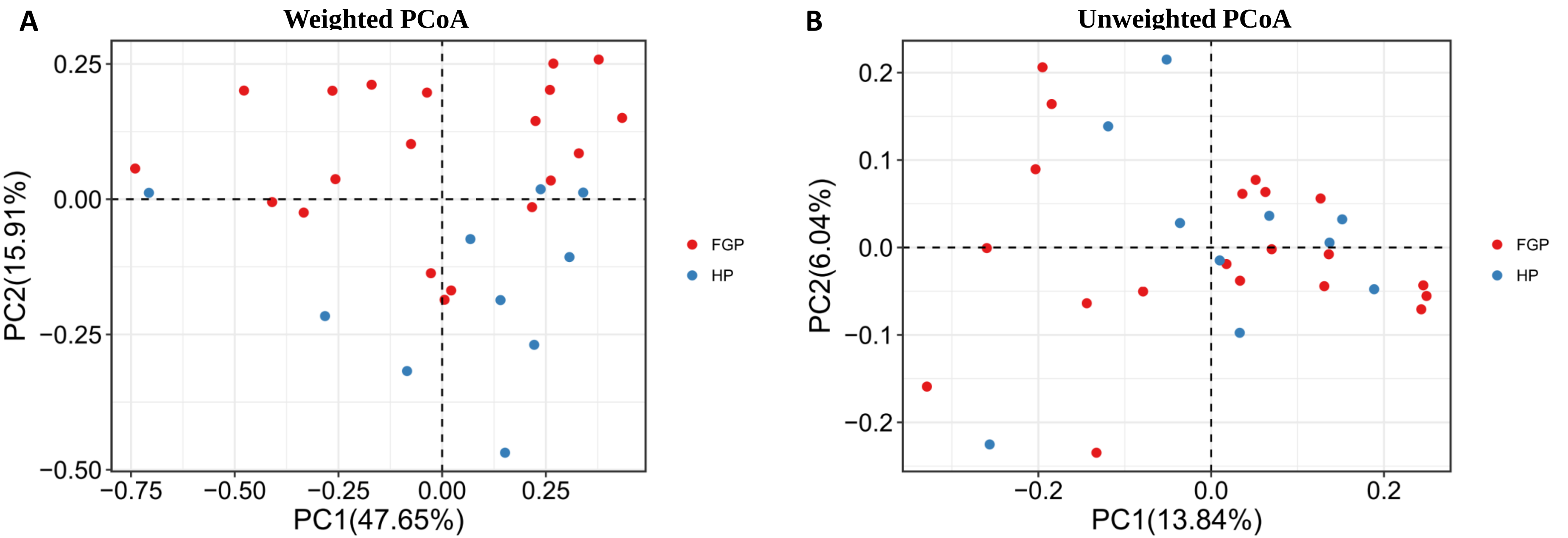

Supplementary figure S3. Beta diversity analysis of gastric bacterial communities in the samples between the FGP and HP groups of only the polyp samples **from the 30 patients with gastric polyposis.** (A) Weighted and (B) unweighted PCoA. FGP: Fundic gland polyp; HP: hyperplastic polyp.

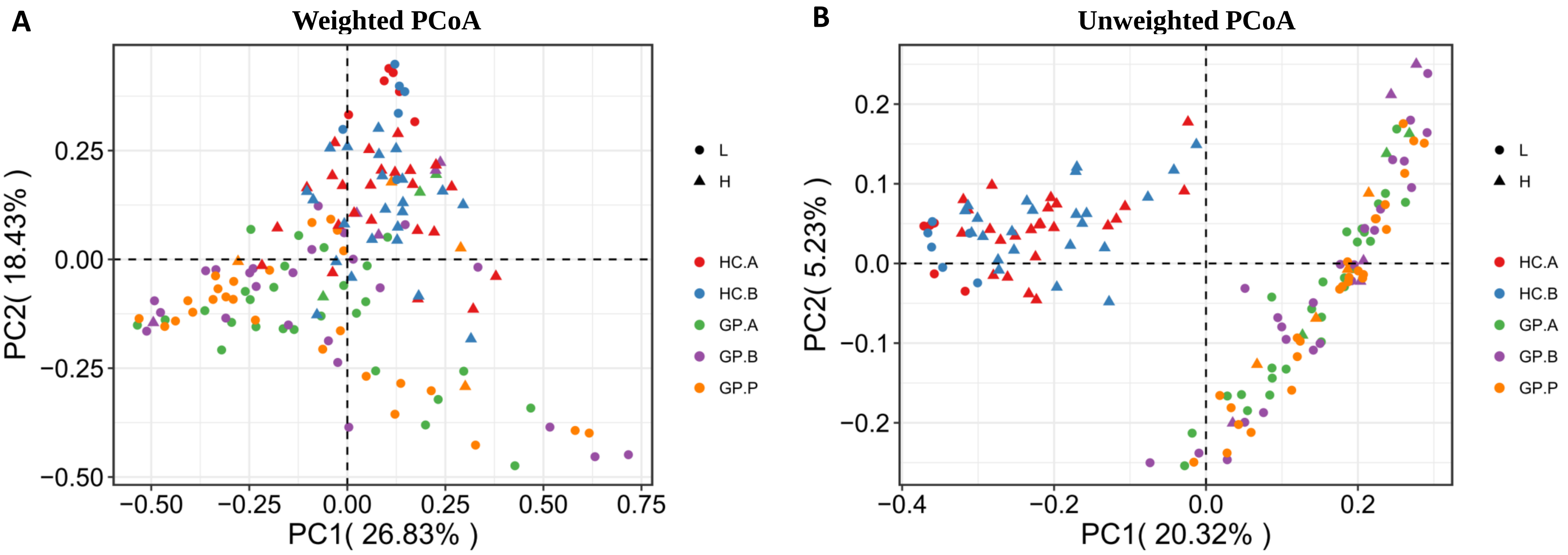

Supplementary figure S4 A, B. Influence of *H. pylori* on the diversity and composition of the bacterial microbiota in the stomach. (A) Weighted and (B) unweighted PCoA of gastric bacterial communities in the samples among HC and GP groups based on the abundance of *H. pylori*. L: low *H. pylori* abundance $<$ 1%; H: high *H. pylori* abundance ≥ 1%. HC: healthy control; GP: gastric polyposis; A: gastric antrum; B: gastric body; P: gastric polyp.

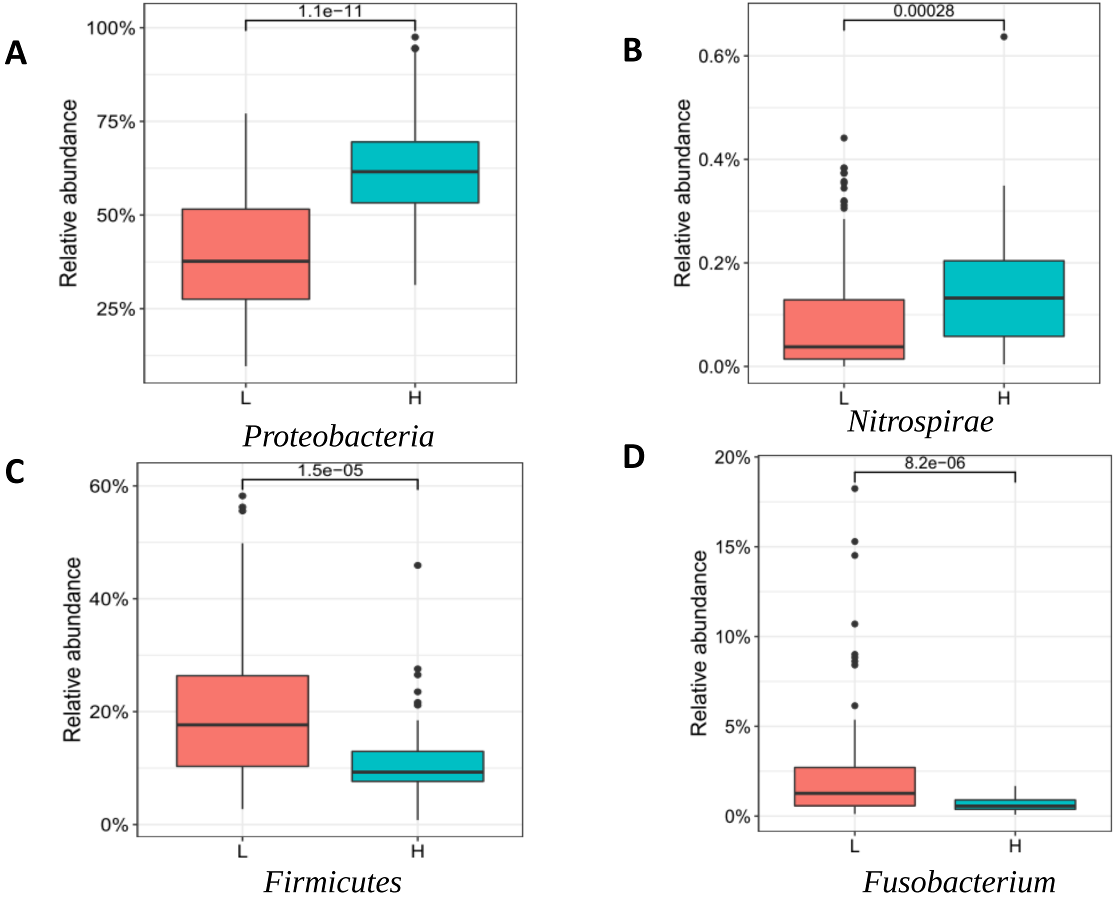

Supplementary figure S5 A-D. Bacteria enriched in *H. pylori*-high group (**A-B**) and *H. pylori*-low group (**C-D**) in phylum level. H: *H. pylori*-high group ; L: *H. pylori*-low group. Statistical significance was determined by Wilcoxon-rank sum test.

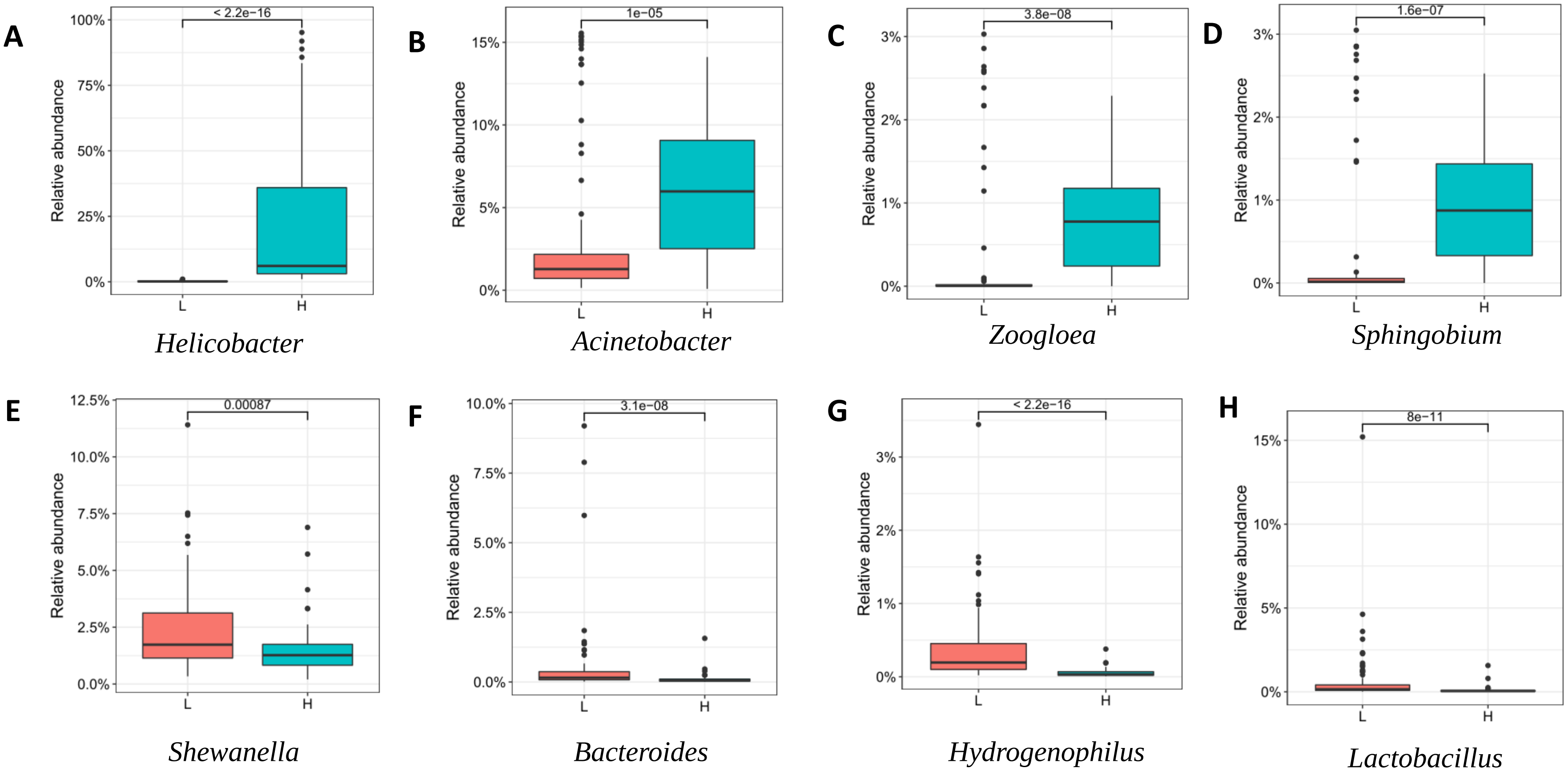

Supplementary figure S6 A-H. Bacteria enriched in *H. pylori*-high group (**A-D**) and *H. pylori*-low group (**E-H**) in genera level. H: *H. pylori*-high group ; L: *H. pylori*-low group. Statistical significance was determined by Wilcoxon-rank sum test.

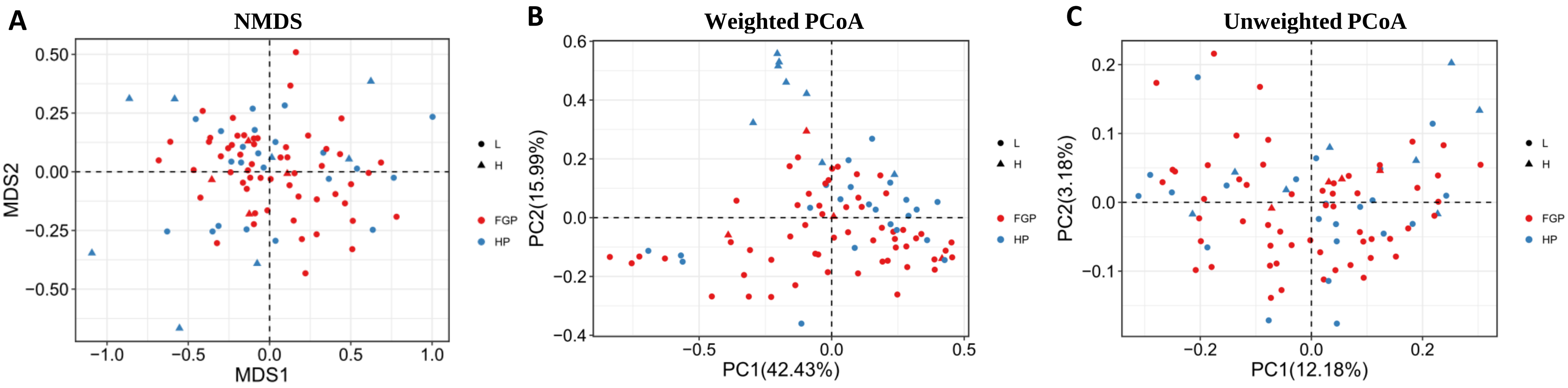

Supplementary figure S7. Influence of *H. pylori* on the distinction of FGP and HP (all samples). (A) NMDS, (B) weighted and (C) unweighted PCoA of gastric bacterial communities of all the samples among the FGP and HP groups based on the abundance of *H. pylori*. L: low *H. pylori* abundance $<$ 1%; H: high *H. pylori* abundance ≥ 1%. FGP: fundic gland polyp; HP: hyperplastic polyp.

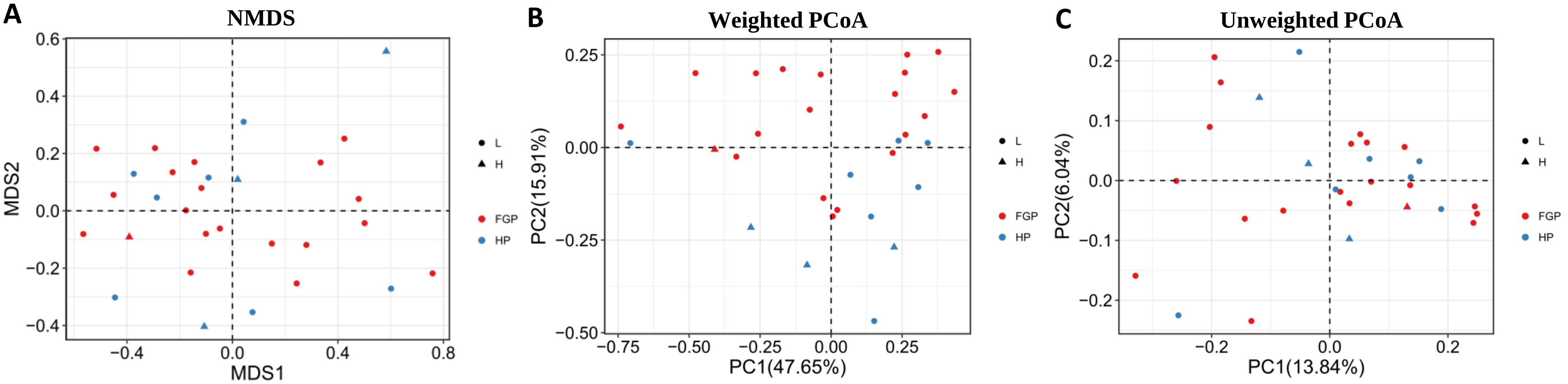

Supplementary figure S8. Influence of *H. pylori* on the distinction of FGP and HP (only polyp samples). (A) NMDS, (B) weighted and (C) unweighted PCoA of gastric bacterial communities of all the samples among the FGP and HP groups based on the abundance of *H. pylori*. L: low *H. pylori* abundance ＜ 1%; H: high *H. pylori* abundance ≥ 1%. FGP: fundic gland polyp; HP: hyperplastic polyp.
